# Prediction of postoperative infectious complications in elderly patients with colorectal cancer: a study based on improved machine learning

**DOI:** 10.1186/s12911-023-02411-0

**Published:** 2024-01-06

**Authors:** Yuan Tian, Rui Li, Guanlong Wang, Kai Xu, Hongxia Li, Lei He

**Affiliations:** 1 Department of Gastrointestinal Surgery, The Third Affiliated Hospital of Anhui Medical University (The first people’s Hospital of Hefei), Hefei, Anhui China; 2 Department of Oncology, The Third Affiliated Hospital of Anhui Medical University (The first people’s Hospital of Hefei), Hefei, Anhui China

**Keywords:** Elderly patients, Colorectal cancer, Postoperative infectious complications, Machine learning, MGA-XGBoost

## Abstract

**Background:**

Infectious complications after colorectal cancer (CRC) surgery increase perioperative mortality and are significantly associated with poor prognosis. We aimed to develop a model for predicting infectious complications after colorectal cancer surgery in elderly patients based on improved machine learning (ML) using inflammatory and nutritional indicators.

**Methods:**

The data of 512 elderly patients with colorectal cancer in the Third Affiliated Hospital of Anhui Medical University from March 2018 to April 2022 were retrospectively collected and randomly divided into a training set and validation set. The optimal cutoff values of NLR (3.80), PLR (238.50), PNI (48.48), LCR (0.52), and LMR (2.46) were determined by receiver operating characteristic (ROC) curve; Six conventional machine learning models were constructed using patient data in the training set: Linear Regression, Random Forest, Support Vector Machine (SVM), BP Neural Network (BP), Light Gradient Boosting Machine (LGBM), Extreme Gradient Boosting (XGBoost) and an improved moderately greedy XGBoost (MGA-XGBoost) model. The performance of the seven models was evaluated by area under the receiver operator characteristic curve, accuracy (ACC), precision, recall, and F1-score of the validation set.

**Results:**

Five hundred twelve cases were included in this study; 125 cases (24%) had postoperative infectious complications. Postoperative infectious complications were notably associated with 10 items features: American Society of Anesthesiologists scores (ASA), operation time, diabetes, presence of stomy, tumor location, NLR, PLR, PNI, LCR, and LMR. MGA-XGBoost reached the highest AUC (0.862) on the validation set, which was the best model for predicting postoperative infectious complications in elderly patients with colorectal cancer. Among the importance of the internal characteristics of the model, LCR accounted for the highest proportion. Conclusions: This study demonstrates for the first time that the MGA-XGBoost model with 10 risk factors might predict postoperative infectious complications in elderly CRC patients.

## Introduction

Due to the progress of population aging and the characteristics of intestinal cell susceptibility in the elderly [1], the proportion of colorectal cancer patients aged 65 or over is as high as 70% [2]. At present, surgery is the cornerstone of colorectal cancer treatment. Relevant data show that the age of patients undergoing intestinal surgery is gradually increasing [3]. However, with the aging process, the organ function and immune function of elderly people over 65 years old decrease, accompanied by more basic diseases. Moreover, elderly patients often have poor nutritional absorption after surgery, poor recovery after invasive treatment, and weak resistance to pathogens, so they are prone to postoperative infection. Therefore, in this study, we pay particular attention to the elderly population to improve the prediction accuracy of this population.

Postoperative infectious complications will increase patient costs, and length of hospital stay, and delay the start time of postoperative adjuvant therapy [4]. It is more important that many pieces of evidence show that postoperative infectious complications are significantly associated with poor prognosis of CRC [5, 6]. If the postoperative infectious complications of elderly patients can be predicted early, the survival quality and prognosis of patients can be improved by the timely use of prophylactic antibiotics and early goal-directed therapy. At present, most of the studies only focus on the effect of individual markers on the prediction of postoperative infection. In this paper, we comprehensively consider the influence of various predictive factors of infectious complications: peripheral blood platelet/ peripheral blood lymphocyte (PLR) [7], peripheral blood lymphocyte/peripheral monocytes (LMR) [8], peripheral blood neutrophil/peripheral blood lymphocyte (NLR) [9] lymphocyte/C-reactive protein (LCR) [10], prognostic nutritional index (PNI) [11] on postoperative infection. It has been reported that these factors can predict the incidence of infectious complications in different types of cancer.

Many researchers have attempted to predict the infectious complications following colorectal surgery by using prediction models, which include various clinicopathological factors. These models rely on traditional statistical analysis, such as logistic risk regression, Cox risk regression, and nomogram. Compared with traditional statistical analysis, the advantage of machine learning is that it can capture complex nonlinear relationships from a series of complex medical data sets, and use data to continuously adapt to improve the accuracy, sensitivity, and specificity of the prediction model [12, 13]. However, some medical personnel may not realize that the traditional ML model has overfitting. Therefore, this study improves the XGBoost algorithm (MGA-XGBoost) based on the moderate greedy (MGA) algorithm to improve the accuracy of the prediction model.

## Methods

### Data sources

In this paper,’ colon cancer ‘and ‘rectal cancer ‘as keywords to retrieve the medical record system of the Third Affiliated Hospital of Anhui Medical University. The clinical data of patients with colorectal cancer confirmed by postoperative pathology after radical operations in gastrointestinal surgery from March 2018 to April 2022 were retrospectively collected. Inclusion criteria:1)Age ≥ 65 years old;2)The patient was diagnosed with colorectal cancer and underwent radical resection of colorectal cancer;3)There was no history of radiotherapy and chemotherapy before the operation, no distant organ metastasis, and the postoperative pathological stage was 0, I, II and III;4)No other malignant tumors were found; 5)Complete clinicopathological data; Excluded criteria:1)Age < 65 years; 2)Incomplete clinical data; 3)Patients with acute and chronic infectious diseases and long-term use of immunosuppressive agents before operation;4)Preoperative radiotherapy and chemotherapy or with distal metastasis;5)Postoperative new non-surgical related diseases;6)Emergency surgery for colorectal cancer with intestinal obstruction;7)Patients who cannot accurately assess postoperative complications without doctor’s advice discharge; Preoperative and intraoperative variables were collected for screening of risk factors. Information on the following 25 variables was obtained: age, sex, body mass index (BMI), ASA, smoking status, Previous comorbidities (chronic lung disease, diabetes), surgical methods, intraoperative blood transfusion, presence of stomy, laboratory examination data: within 7 days before surgery(lymphocytes, C-reactive protein, soterocyte, albumin, monocytes, white blood cells, hematocrit, international normalized ratio, fibrinogen, total bilirubin, direct bilirubin, aspartate aminotransferase (AST), blood urea nitrogen (BUN), creatinine, uric acid, Na + 、Ca+), Tumor information: pathological T-stage(T-stage), pathological N-stage (N-stage), pathological stage, tumor location, tumor size; Operation information: intraoperative bleeding, operation time.

### Postoperative infection

The common postoperative infectious complications were observed, including respiratory and pulmonary infection, incision infection, anastomotic leakage, abdominal abscess, urinary tract infection, etc. The diagnostic criteria of infection refer to the corresponding guidelines and standard references [5, 14, 15]. Briefly, as follows:1) Incision infection: Skin and subcutaneous tissue infection within 30 days after surgery, wound redness, swelling, heat, pain, local incision drainage pus;2) anastomotic leakage: Clinical signs of peritonitis such as tenderness, rebound pain, and muscle tension were observed. Color Doppler ultrasound showed gas and liquid around the anastomosis, or CT showed anastomotic disconnection;3) Abdominal abscess: Abdominal space infection occurred within 30 days after the operation, manifested as abdominal pain, persistent fever, and other symptoms, confirmed by puncture or B-ultrasound and improved after surgical drainage or anti-infective treatment;4) Uinary tract infection: Cystitis and urethritis occurred within 30 days after the operation. Bladder irritation symptoms such as frequent urination, urgency, and dysuria occurred clinically. A routine urine examination showed pyuria and hematuria. Pathogenic bacteria were cultured in urine;5) Pulmonary infection: The patient presented with body temperature > 38.0 °C, elevated white blood cell count, cough, expectoration, and other clinical symptoms. Dry and wet rales were heard in the lungs, and a chest X-ray showed new invasive lesions.

### Conventional statistical analysis

SPSS24.0 software was used to process and analyze the data. The optimal cutoff values of NLR, PLR, PNI, LCR, and LMR were determined by the receiver operating characteristic curve, as shown in Table 1. There is no uniform standard for the study of the five optimal cut-off values determined by AUC. The cut-off values of PNI ranged from 40.1 [16] to 51.26 [17], the cut-off values of LCR ranged from 0.34 [18] to 0.84 [19], the cut-off values of NLR ranged from 1.93 [17] to 4.8 [20], the cut-off values of PLR ranged from 190.83 [21] to 645.22 [17], and the cut-off values of LMR ranged from 2 [22] to 3.6 [23], which were consistent with the results of this study. In the univariate analysis, Continuous variables (such as Body Mass Index) were reported as mean ± standard deviation and analyzed using the U test to assess the significance level between the infected group and the non-infected group. Count data in univariate analysis were expressed by rate or the number of cases, and the *χ*2 test was used between groups. Factors with statistical significance in single factors were included in the machine learning model. *P* < 0.05 was considered statistically significant. We continuous variables were normalized based on the mean and SD of the training set. Categorical variables were encoded into binary variable, 1 represents having an incident, 0 represents not having an incident. Gender was also encoded, 1 represents male, 0 represents female. Overfitting may occur in the process of model training, thus destroying the performance of the model. Therefore, we first perform single factor analysis to filter out features that are not statistically significant, and then introduce the recursive feature elimination (RFE) method of random forest. This method first trains all features, then recursively removes the least important features, and selects the feature set with the highest recall score [24].

**Table 1:** The best cut-off values of the five indicators

Inflammatory Markers	ROC (95%CI)	Recall	Specificity	Optimal cut-off Value
NLR	0.656(0.583–0.730)	0.474	0.780	3.80
PLR	0.624(0.548–0.700)	0.434	0.818	238.50
PNI	0.606(0.532–0.679)	0.829	0.343	48.48
LCR	0.802(0.742–0.861)	0.842	0.742	0.52
LMR	0.674(0.587–0.761)	0.617	0.740	2.46

### Machine learning

In this paper, python3.9 was used to construct various machine learning models (Linear Regression, Random Forest, SVM, BP, LGBM, XGBoost) to predict postoperative infectious complications in elderly patients with colorectal cancer. Except for XGBoost, the other five models are built by installing the scikit-learn package in python3.9. The data of 512 elderly patients were randomly divided into a 70% training set and a 30% validation set. The training set data is used to develop the prediction model, and the validation set data is used to verify the performance of the model. The performance of the model was evaluated by the AUC, ACC, recall, F1-score, and precision.

### Development of optimization algorithm

The use of the XGBoost model often faces two major problems:1) When the XGBoost model is used for prediction, there are many parameters to be adjusted, and the process of parameter adjustment is tedious. It is difficult to select the best parameters for the current problem;2) The XGBoost model applied to the idea of Gradient Boosting has the risk of overfitting; Therefore, this paper uses Greedy Algorithm (GA) to adjust the parameters; However, the GA algorithm also has some shortcomings in the context of the current problem. For example, the result of the previous iteration will directly affect the result of the next iteration, resulting in a fallacy. Then the greedy algorithm will cause a large error in the final result. Therefore, this paper proposes a Moderate Greedy Algorithm (MGA) to remedy and correct this. MGA is consistent with GA in solving the problem and will make a better choice in the current state and gradually construct the optimal solution. MGA is actually to introduce the principle of moderation based on GA thought, restrain the greedy range, avoid excessive greed, and lead to the accumulation of errors, resulting in a large error in the final result. The optimal results can be obtained by selecting the appropriate moderate principle, and the weighted ensemble learning method is used to increase its robustness.

In this paper, the MGA algorithm is used to adjust the max_depth, min_child_weight, gamma, subsample, colsample_bytree, reg_alpha, reg_lambda parameters of XGBoost. The parameters are grouped in a greedy idea and optimized step by step, and e-ach time does not only depend on the optimal parameter subset but select several optimal parameter subsets. If the seven parameters are optimized by grid search, it not only has a large amount of calculation, but also limits the range of each parameter. Therefore, we use a greedy method to group the parameters and optimize them step by step, and each time we do not only depend on the optimal parameter subset, but also select several optimal parameter subsets (so the algorithm is called ‘MGA’). The main operation details are shown in Table 2.

**Table 2:** The value process of MGA

Parameter adjustment order	Parameter name	Parameter adjustment standard
①	max_depth, min_child_weight	Choose the best two groups
②	gamma	Choose the best two groups
③	subsample, colsample_bytree	Choose the best two groups
④	reg_alpha、reg_lambda	Choose the best one groups
⑤	learning_rate	Choose the best one groups
⑥	num_boost_round	The selection of num _ boost _ round is to make training _ AUC > 0.9995

The value range of parameter adjustment is shown in Table 3:

**Table 3:** Range of XGBoost parameters

Parameter name	Value ranges
max_depth	[3–8]
min_child_weight	[1–6]
gamma	[i/10.0 for i in range (0,5)]
subsample	[i/10.0 for i in range (6,10)]
colsample_bytree	[i/10.0 for i in range (6,10)]
reg_alpha	[0,1e-8,1e-7,1e-6,1e-5,1e-4,1e-3,1e-2,0.1,1,100]
reg_lambda	[0,1e-6,1e-5,1e-4,1e-3,1,10,100]

Based on the idea of greedy algorithm, we divide the parameter adjustment process of XGBoost into six steps. Under the condition of local optimal parameters obtained after each step of parameter adjustment, the next step is to optimize other parameters. And so on until all parameters are adjusted.

The main idea of boosting algorithm is to combine multiple weak learners with high deviation to reduce the overall deviation and form a strong learner. we worry that if a single XGBoost is used, it will perform poorly in modeling. In order to avoid the risk of overfitting due to inconsistent data distribution and small data sample size, we use the integration of XGBoost to increase the robustness of the model. In the process of parameter adjustment, not only the optimal set of parameters is taken, but several sets of better parameter models are selected. Steps of parameter adjustment:

① First adjust the two sets of parameters of max_depth and min_child_weight, and select the two sets of parameters with the best score.
② Secondly, the gamma parameter is adjusted to retain the optimal two sets of data.
③ Then adjust the two sets of parameters of subsample and colsample_bytree, and select the optimal two sets of data.
④ Then, the parameters of the two sets of regular coefficients reg_alpha and reg_lambda are adjusted to select the optimal set of data.
⑤ Therefore, there are now 2*2*2*1 = 8 sets of data. Finally, the parameters of learning_rate and num_boost_round are adjusted to select the optimal set of parameters.

Here in the tuning step also consider divided into different ‘step group ‘, The so-called step group is the nine parameters listed above, which can be randomly divided into several steps, and adjust one or two parameters in each step. For example, the above five-step adjustment can be used as a step group; “the first step: max_depth; the second step: min_child_weight; the third step: gamma; step 4: subsample, colsample_bytree; step 5: reg_alpha, reg_lambda; the sixth step: learning_rate, num_boost_round”, such parameter group adjustment can be said to be another “step group”. In summary, I finally got a total of 8 sets of optimal XGBoost experimental parameters as follows (Table 4):

**Table 4:** 8 groups of XGBoost model parameter results

max_depth	min_child_weight	gamma	subsample	colsample_bytree	reg_alpha	reg_lambda	learning_rate	num_boost_round
3	3	0.2	0.9	0.8	1.00E-05	1	0.01	5000
3	3	0.2	0.8	0.8	1.00E-05	1	0.01	5000
3	3	0.4	0.9	0.5	1.00E-05	1	0.01	5000
3	3	0.4	0.95	0.5	1.00E-05	1	0.01	5000
7	4	0	0.9	0.95	1.00E-05	1	0.01	1000
7	4	0	0.95	0.95	1.00E-05	1	0.01	1000
7	4	0.1	0.9	0.3	1.00E-05	1	0.01	1000
7	4	0.1	0.95	0.4	1.00E-05	1	0.01	2000

The eight sets of XGBoost experimental parameter models obtained by the above methods are compared and sorted according to the optimal and sub-priority of the parameters, and then weighted ensemble learning is performed. The optimal allocation weight is 2/3, and the suboptimal allocation weight is 1/3. The number of iterations is set to 500. Therefore, the proportion of the eight groups of parametric models obtained is 0.296, 0.148, 0.148, 0.074, 0.148, 0.074,0.074, 0.074, 0.038(See the source code for details: https://github.com/ahmutty/MGAXG. Therefore, the final MGA-XGBoost model is Model = 0.296*model1 + 0.148*model2 + 0.148*model3 + 0.074*model4 + 0.148*model5 + 0.074*model6 + 0.074*model7 + 0.038*model8.

## Results

### Patient characteristics

From March 2018 to April 2022,563 elderly patients underwent radical resection of colorectal cancer in the gastrointestinal surgery department of our hospital. After exclusion and inclusion criteria screening,512 patients were included in the study. In this completed data set, no variables had missing percentage higher than 1%. We employed mean imputation, which imputed missing value with the mean of each feature, to fill in missing values. Patients with postoperative infectious complications accounted for 24% (*n* = 125), 70% (*n* = 358) in the training sets, and 30% (*n* = 154) in the validation set. There were 295 male patients (57.62%) and 217 female patients (42.38%). The characteristics of the data set are shown in Table 5.

**Table 5:** characteristics of the study patients(*n* = 512)

Risk factor	Mean	SD	Min	P_25_	P_50_	P_75_	Max
BMI (kg/m2)	22.13	3.04	13.8	19.91	22.08	24	31.59
Operation Time(h)	3.59	1.24	0.75	2.75	3.39	4.33	9.17
Blood loss (ml)	128.17	168.87	20	2000	50	100	100
NLR	4.11	5.26	0.38	1.79	2.6	4.08	66.73
PLR	186.38	127.27	9.83	107.09	150.32	218.59	855.26
PNI	45.06	7.39	21.57	41.32	46.01	49.8	69.7
LCR	0.94	1.09	0.02	0.24	0.64	1.04	7.4
LMR	3.65	5.89	0.28	2.24	3.11	4.02	8.76

### Feature selection using univariate and recursive feature elimination methods

**Fig. 1:**
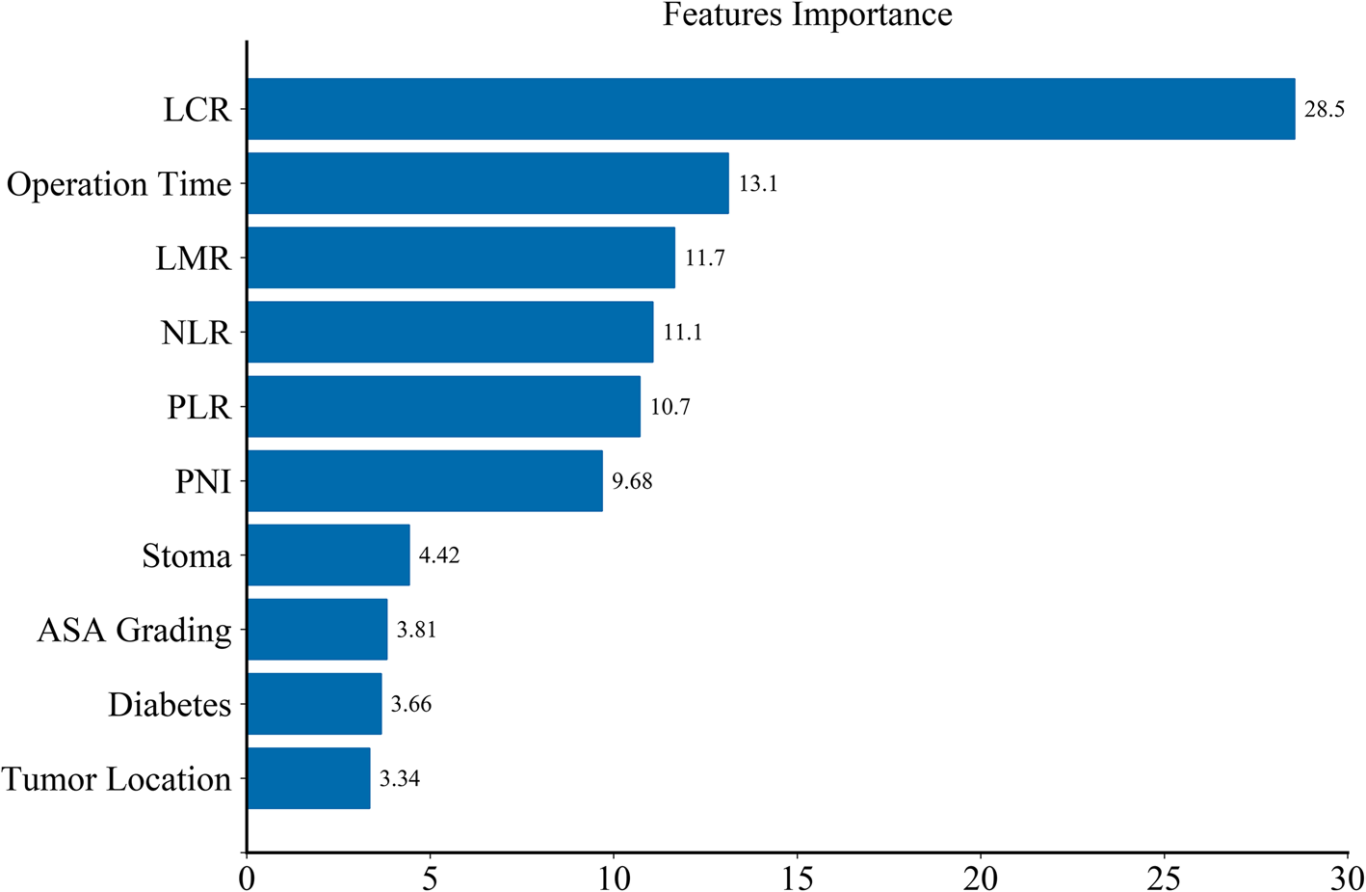
Feature importance ranking of the selected 10 features illustrated by random forest

To better understand the data characteristics of the model, the patients were divided into an infected group and a non-infected group according to the training set and validation set, and then the data were analyzed by single factor analysis. Since less relevant features may have a negative impact on the performance of machine learning models, we further use the recursive feature elimination (RFE) method to select features and rank the importance of features. The univariate and RFE methods are used for feature selection to reduce 36 features to 10 features. These 10 features were ASA, operation time, diabetes, presence of stomy, tumor location, NLR, PLR, PNI, LCR, and LMR (*P* < 0.05). The results of single factor analysis are shown in Table 6, and the feature ranking of RFE method is shown in Fig. 1.

**Table 6:** Data characteristics analysis of the infected group and non-infected group(*n* = 512)

Variables	Training sets			Validation set		
	Infection group (*n* = 87)	Non-infected group (*n* = 271)	*P*	Infection group (*n* = 38)	Non-infected group (*n* = 116)	*P*
Gender			0.06			0.09
Male	57(0.66)	147(0.54)		18(0.47)	73(0.63)	
Female	30(0.34)	124(0.46)		20(0.53)	43(0.37)	
Body Mass Index (kg/m^2^)	22.02(3.54)	21.88(3.56)	0.769	21.33(3.66)	21.89(3.58)	0.458
ASA			< 0.001			0.001
I	10(0.11)	13(0.05)		6(0.16)	10(0.09)	
II	34(0.39)	179(0.66)		17(0.45)	88(0.76)	
III	43(0.50)	79(0.29)		15(0.39)	18(0.15)	
Tumor size (cm)	4.57(1.78)	4.58(1.94)	0.987	4.86(2.16)	4.93(1.87)	0.865
T-stage						
T1–2	26(0.30)	78(0.29)	0.844	14(0.37)	39(0.34)	0.717
T3–4	61(0.70)	193(0.71)		24(0.63)	77(0.66)	
N-stage						
N-	57(0.66)	159(0.59)	0.256	28(0.74)	81(0.70)	0.650
N+	30(0.34)	112(0.41)		10(0.26)	35(0.30)	
TNM stage			0.282			0.73
I	22(0.25)	65 (0.24)		9(0.24)	35 (0.30)	
II	36(0.41)	91 (0.34)		16 (0.42)	43(0.37)	
III	29 (0.34)	115 (0.42)		13 (0.34)	38 (0.33)	
Operative time (h)	4.12(1.38)	3.42(1.18)	< 0.001	4.07(1.73)	3.38(1.14)	0.008
Blood loss (ml)	128.42(143.36)	109.53(103.54)	0.43	187.74(235.74)	143.21(163.92)	023
Smoking			0.847			0.707
Yes	5(0.06)	15(0.06)		4(0.11)	8(0.07)	
No	82(0.94)	256(0.94)		34(0.89)	108(0.93)	
Chronic lung disease			0.007			0.003
Yes	19(0.22)	20(0.07)		11(0.29)	11(0.09)	
No	68(0.78)	251(0.93)		27(0.71)	105(0.91)	
Hypertension			0.458			0.306
Yes	33(0.38)	115(0.42)		20(0.53)	50(0.43)	
No	54(0.62)	156(0.58)		18(0.47)	66(0.57)	
Diabetes			< 0.001			0.003
Yes	19(0.22)	18(0.07)		10(0.26)	8(0.07)	
No	68(0.78)	253(0.93)		28(0.74)	108(0.93)	
Surgical procedure			0.301			0.121
Laparoscopic	18(0.21)	71(0.26)		8(0.21)	40(0.34)	
Open	69(0.79)	200(0.74)		30(0.79)	76(0.66)	
Stomy			0.006			0.033
Yes	34(0.39)	65(0.24)		16(0.42)	28(0.24)	
No	53(0.61)	206(0.76)		22(0.58)	88(0.76)	
Location			< 0.001			< 0.001
Rectum	21(0.24)	126(0.46)		8(0.21)	62(0.53)	
Colon	66(0.76)	145(0.54)		30(0.79)	54(0.47)	
Blood transfusion (mL)			0.003			0.012
Yes	26(0.30)	42(0.15)		14(0.37)	20(0.17)	
No	61(0.70)	229(0.85)		24(0.63)	96(0.83)	
WBC (10^9^/L)	6.23(2.26)	6.53(2.31)	0.300	5.76(2.05)	6.80(2.30)	0.027
Lymphocytes (10^9^/L)	1.50(0.91)	1.32(0.50)	0.110	1.31(0.49)	1.38(0.49)	0.529
Hematocrit	36.14(5.87)	35.23(5.54)	0.631	35.69(6.31)	36.16(5.72)	0.512
Soterocyte (10^9^/L)	216.25(84.76)	219.47(86.32)	0.875	218.36(78.65)	220.47(80.25)	0.896
International normalized ratio	1.05(0.14)	1.06(0.10)	0.681	1.05(0.08)	1.05(0.15)	0.762
Fibrinogen (ug/mL)	3.32(1.02)	3.45(1.16)	0.632	3.32(1.12)	3.47(1.03)	0.613
Total bilirubin (μmol/L)	14.63(8.17)	13.69(8.15)	0.156	13.24(6.54)	13.65(6.67)	0.778
Direct bilirubin(μmol/L)	5.28(3.45)	5.49(3.48)	0.721	4.42(3.25)	4.71(3.18)	0.598
AST (U/L)	23.08(12.65)	20.27(12.87)	0.241	23.26(12.63)	21.08(10.32)	0.268
BUN (mmol/L)	6.43(2.87)	5.87(2.97)	0.367	6.14(3.08)	5.87(2.18)	0.674
Cr(μmol/L)	75.65(21.21)	70.25(21.36)	0.214	78.58(23.64)	70.54(20.97)	0.074
Uric acid (μmol/L)	315.24(106.54)	301.02(143.26)	0.645	307.87(99.64)	293.57(136.57)	0.524
Serum sodium (mmol/L)	141.05(3.62)	141.71(3.02)	0.241	141.08(3.65)	141.94(2.87)	0.235
Serum calcium (mmol/L)	2.22(0.19)	2.24(0.18)	0.487	2.22(0.19)	2.25(0.19)	0.412
NLR			< 0.001			0.002
≥ 3.80	39(0.45)	60(0.22)		17(0.45)	30(0.26)	
< 3.80	48(0.55)	211(0.78)		21(0.55)	86(0.74)	
PLR			< 0.001			0.045
≥ 238.50	37(0.43)	50(0.18)		14(0.37)	24(0.21)	
< 238.50	50(0.57)	221(0.82)		24(0.63)	92(0.79)	
PNI			0.025			0.011
≥ 48.48	17(0.20)	87(0.32)		6(0.16)	44(0.38)	
< 48.48	70(0.80)	184(0.68)		32(0.84)	72(0.62)	
LCR			< 0.001			< 0.001
≥ 0.52	6(0.07)	195(0.72)		12(0.32)	94(0.81)	
< 0.52	81(0.93)	76(0.28)		26(0.68)	22(0.19)	
LMR			< 0.001			< 0.001
≥ 2.46	32(0.37)	201(0.74)		16(0.42)	93(0.80)	
< 2.46	55(0.63)	70(0.26)		22(0.58)	23(0.20)

### Correlation analysis between risk factors

**Fig. 2:**
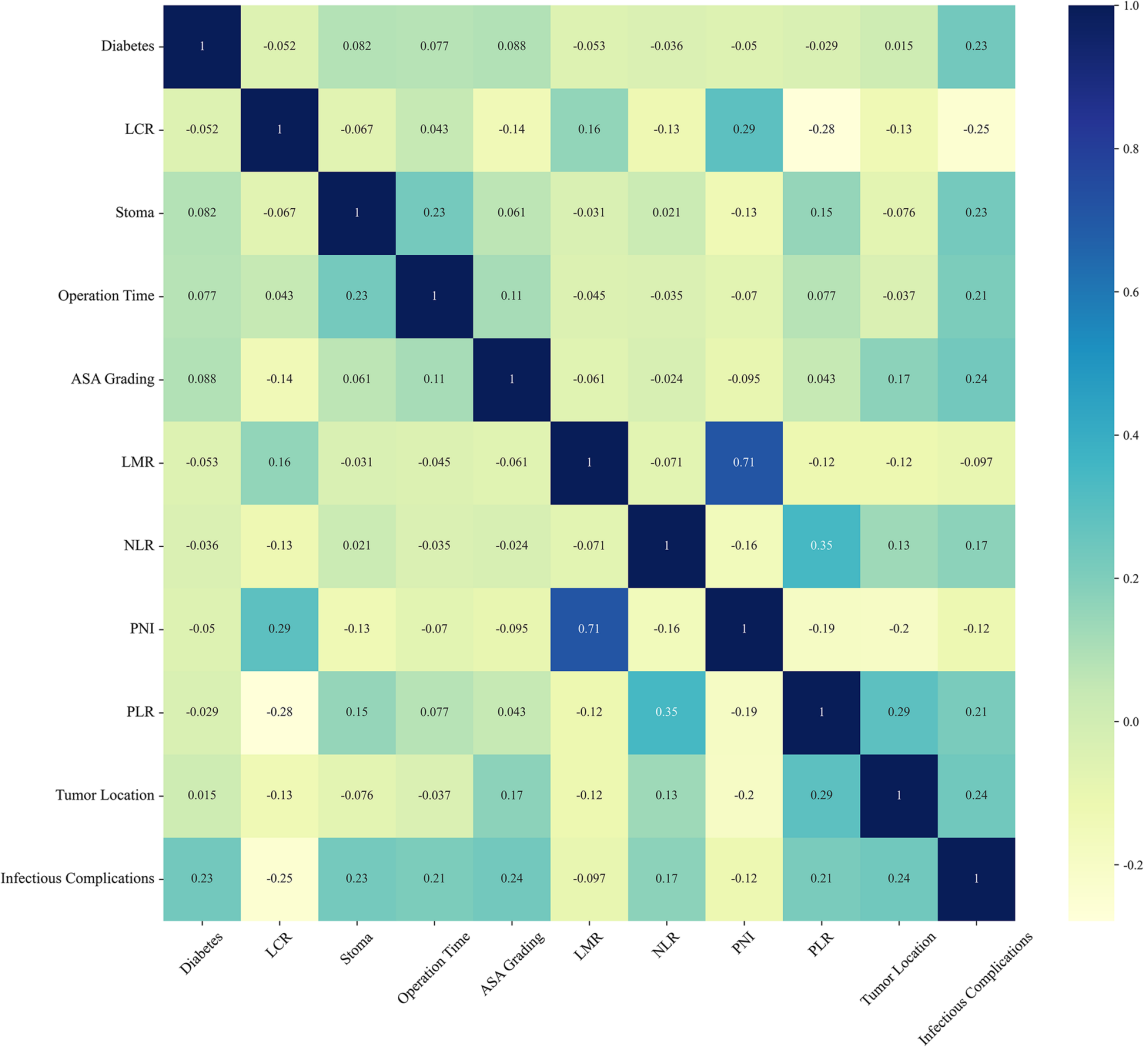
Correlation analysis between risk factors

To better see whether there is a correlation between risk factors, this paper analyzes the correlation of statistically significant indicators in RFE methods. The results showed that there was a high correlation between PNI and LMR (0.71)、NLR and PLR (0.35). The detailed results are shown in Fig. 2.

### Performance evaluation of machine learning models for predicting postoperative infectious complications

**Fig. 3:**
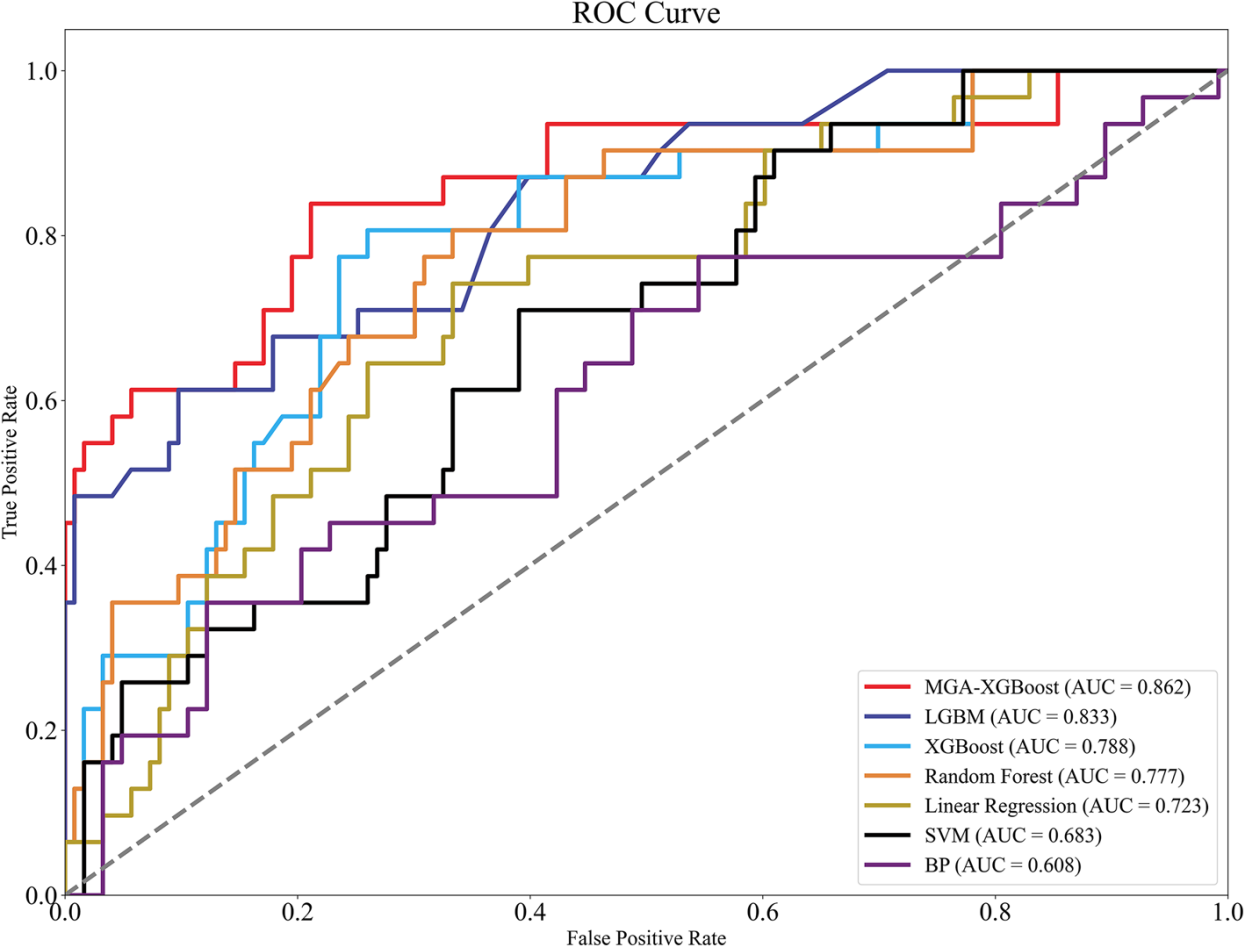
ROC curve for predicting postoperative infectious complications on the validation set

To evaluate the predictive effect of seven machine learning models on postoperative infectious complications in elderly patients. The results showed that the AUC value of the MGA-XGBoost prediction model was the highest (0.862), and Linear Regression, SVM, and BP all showed general predictive ability (the AUC range was 0.6 ~ 0.73). The AUC value of each model is shown in Fig. 3.

In addition to AUC, this paper also introduces ACC, Recall, F1-score, and Precision to evaluate the performance of various prediction models. It can be seen from Table 7 that MGA-XGBoost, LGBM and XGBoost all show good accuracy and precision.

**Table 7:** The performance of 7 ML models in the validation set

Model	AUC	ACC	Recall	F1-score	Precision
MGA-XGBoost	0.862	0.877	0.679	0.704	0.731
XGBoost	0.833	0.844	0.618	0.66	0.708
LGBM	0.788	0.786	0.532	0.581	0.64
Random Forest	0.777	0.773	0.581	0.621	0.668
Linear Regression	0.723	0.731	0.29	0.353	0.45
SVM	0.683	0.74	0.323	0.333	0.345
BP	0.608	0.714	0.418	0.271	0.2

### Feature importance analysis of MGA-XGBoost model

In this paper, the importance of internal features in the verification data set of the MGA-XGBoost prediction model with the highest accuracy is visually displayed by three methods of cover, weight and gain. Visualized mathematical publicity is:

$$S=\left(\frac{i_{cover}}{\sum_i cover}+\frac{i_{weight}}{\sum_i weight}+\frac{i_{gain}}{\sum_i gain}\right)\times 100$$

**Fig. 4:**
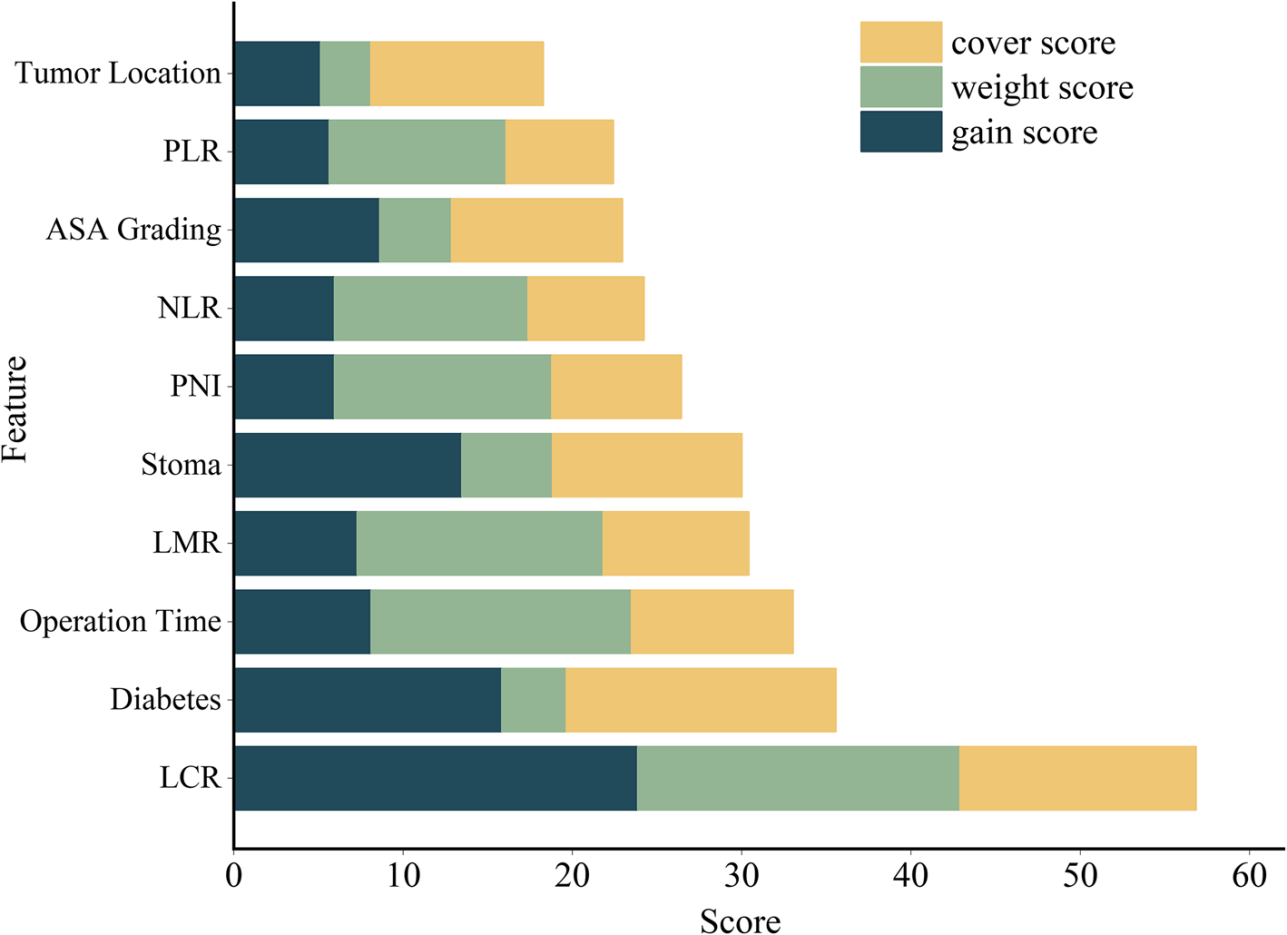
Feature importance analysis of MGA-XGBoost model in the validation set

Where *S* is the total score of the three methods of each feature, *i* is the score of each independent feature, *i*_*cover*_, *i*_*weigh*_*t* and *i*_*gain*_ are the scores of each independent feature.) As shown in Fig. 4, LCR, diabetes and operation time ranked first, second and third respectively.

## Discussion

This research based on clinical data and machine learning methods has the following main contributions:1) The first study found that 10 factors were significantly associated with infectious complications after colon cancer surgery: ASA, operation time, diabetes, tumor location, presence of stomy, NLR, PLR, PNI, LCR, and LMR;2) The second study constructed a conventional predictive model for postoperative infectious complications in elderly patients with colorectal cancer. The results showed that the LGBM model performed best in predicting postoperative infection compared with the other five machine learning models. The AUC was 0.833, the accuracy was 0.844, and the precision was 0.708;3) The third research work is extended based on the second work, focusing on improving the XGBoost model to improve the accuracy of the model. The results show that the MGA-XGBoost prediction model has the highest AUC value (0.862), the accuracy is 0.877, and the precision is 0.731, showing great potential for future application in the field of intelligent medical care;4) The fourth research work visualizes the importance of internal features of the MGA-XGBoost prediction model, overcomes the shortcomings of opaque and unexplainable machine learning, and greatly improves the future clinical application prospects of machine learning;5) Finally, in the case of early diagnosis of postoperative infection by the model, antibiotics can be used in time for treatment, rather than treatment based on late symptoms and clinical deterioration. Avoid unnecessary and excessive use of antibiotics in low-risk patients. At the same time, postoperative care should be strengthened for high-risk patients, such as actively encouraging patient activity, promoting sputum coughing, and improving clinical outcomes in elderly patients.

The role of systemic inflammatory response and nutritional status in cancer patients is increasingly recognized [25]. For example, systemic inflammatory response indicators and nutritional indicators can be used to predict infectious complications after malignant tumor surgery [11, 26]. Okugawa [10] found that low preoperative LCR was an independent risk factor for surgical site infection in patients with colorectal cancer. Because cancer status usually activates systemic inflammatory responses, invasive surgery triggers abnormally enhanced inflammatory responses that reduce patient immunity [27]. Consistent with our study, preoperative NLR and PLR levels increased, and LCR and LMR levels decreased, suggesting a higher risk of postoperative infectious complications. It is worth noting that LCR ranks first in the importance ranking of internal features of MGA-XGBoost model. Okita [28] pointed out that low PNI may be a significant predictor of postoperative infectious complications in patients with ulcerative colitis undergoing proctectomy with ileal pouch-anal anastomosis. Cancer patients occasionally have impaired nutritional intake during the perioperative period [29]. Malnutrition can also lead to the decline of immune function in cancer patients [30], especially hypoproteinemia has a significant effect on humoral immunity, which can cause pathogen translocation, conditional pathogen transformation, and fungal reproduction [31]. Studies have shown that immune nutrition and special enteral formula can reduce the incidence of postoperative infectious complications in patients with colorectal cancer surgery [32]. The results of this study showed that with preoperative PNI < 48.48, the incidence of postoperative infectious complications increased. Therefore, this study showed that inflammatory response and nutritional indicators were significantly associated with postoperative infection. At the same time, this study determined five comprehensive inflammatory indicators related to postoperative infection of colorectal cancer by single factor analysis and RFE method. According to different literature reports, these risk factors were significantly associated with postoperative infection [33, 34]. In the era of rapid rehabilitation surgery, it is important to use these markers for early prediction of infection, and early diagnosis to avoid readmission and reduce medical costs.

ML refers to the iterative and automatic optimization of mathematical models to gradually and accurately fit available data [35]. There are thousands of machine learning algorithms, but each model has its limitations and the best algorithm is uncertain in different situations [36]. The best model usually depends on the sample data set and analysis purpose in a specific scenario [37]. For example, the BP model in this study has the lowest accuracy, which may be because BP transforms the characteristics of all problems into numbers and all reasoning into numerical calculations, resulting in the loss of information in its results [38]. Therefore, in this study, we calculated the prediction accuracy of six conventional machine learning models and compared their performance, among which the LGBM model showed the best prediction ability. LGBM prevents the model from falling into the local optimal solution by pruning and uses the second derivative to use the sampling method in each iteration to prevent overfitting [39]. Therefore, LGBM has the best overall performance in the conventional machine learning model for predicting postoperative infection of colorectal cancer, with an AUC of 0.833, an accuracy of 0.844, and a precision of 0.708.

Most clinicians usually use standard statistical software packages (such as R) to develop some machine learning methods, but standard software packages cannot make up for the shortcomings of machine learning itself. For example, XGBoost performs well in various ML competitions, but it usually has problems with many parameters and cumbersome adjustments. Therefore, some scholars have studied the improvement of XGBoost. In 2021, Peng [40] constructed a new model for predicting hypertension based on hybrid feature selection and standard XGBoost. The new model is about 7% higher than the AUC and the accuracy of the model is without improvement. Zhang [41] proposed a GA-XGBoost model for diabetes risk prediction. The experimental results show that the prediction accuracy of the GA-XGBoost model is better than that of linear regression, decision tree, support vector machine, and neural network, and the parameter adjustment time is less than that of grid search and random walk. In this study, Python3.9 uses the greedy idea to group the parameters and tune them step by step. Each time several parameter subsets are selected, and the final model is obtained by weighting. After training on 70% of the full data set, MGA-XGBoost increased AUC by 7.4% in the 30% data set test. Therefore, the improved XGBoost model established in this study can help clinicians make the best prediction. This study shows that advances in artificial intelligence and machine learning will positively improve the performance of clinical predictive models.

Although complex algorithms such as XGBoost, support vector machine, and artificial neural network are increasingly popular and widely used in predictive modeling, they are based on a ‘black box ‘design and are difficult to explain and apply in clinical practice [42]. Clinicians should require the transparency and interpretability of the algorithm so that artificial intelligence can be responsible for its predictions and recommendations. However, the improvement of model interpretability cannot be at the expense of accuracy. Our main goal is to construct a more accurate, interpretable, and robust ML model for postoperative infection in elderly patients with colorectal cancer. Therefore, this study the importance of internal features in the verification data set of the MGA-XGBoost prediction model with the highest accuracy is visually displayed by three methods of cover, weight and gain. By opening the internal structure of the MGA-XGBoost model, the priority of these features in this study is distinguished. This method is superior to other previously published opaque machine learning models. This provides an important basis for the clinical perioperative management of elderly patients. For controllable risk factors, clinicians can consider taking intervention measures to solve these risk factors or control them within a certain range before surgery to optimize the patient’s condition.

In this study, the interior of the MGA-XGBoost model shows the importance of blood glucose indicators. In the comparative correlation analysis, the correlation between blood glucose and postoperative infectious complications was only 0.23, so it may be missed in routine analysis. Postoperative hyperglycemia is a common perioperative stress response [43]. Marks [44] believed that perioperative blood glucose in diabetic patients should be stable at 6.67–10.0 mmol/L, and blood glucose greater than 13.9 mmol/L and less than 4.8 mmol/L are unfavorable to patients. At the same time, Nakamura et al. [45] pointed out that even under strict perioperative blood glucose control, diabetes is directly related to the increased risk of surgical site infection. It shows that there is an internal relationship between diabetes and surgical partial infection, not just diabetes-related hyperglycemia. It may be due to metabolic disorders such as sugar and protein in diabetic patients, resulting in reduced white blood cell bactericidal capacity and reduced production of immunoglobulins and antibodies, resulting in low immunity. In addition, elderly patients with a longer duration of diabetes are prone to vascular neuropathy, resulting in slow blood flow and reduced tissue oxygen supply, which is conducive to the growth of fungi and anaerobic bacteria, so they are more prone to postoperative infection than non-diabetic patients. Therefore, medical staff should strictly control the blood glucose level of diabetic patients, and continue to use insulin during the perioperative period to avoid excessive blood glucose fluctuations.

In the study of inflammatory response indicators, Okugawa [10] compared the predictive ability of LCR, CAR, NLR, PLR, and other inflammatory indicators. The results showed that LCR had the highest correlation with colorectal cancer recurrence and was a more reliable biomarker. It may be because preoperative CRP is associated with lymphopenia and T lymphocyte reaction cell damage in patients with colorectal cancer [46], and lymphocytes play a key role in the host’s cytotoxic immune response to tumors, which impairs cell-mediated immunity in patients with colorectal cancer. In the MGA-XGBoost model, LCR is the best predictor of postoperative infection in colorectal cancer compared with other inflammatory indicators.

For patients with longer operation time, the operation will increase the exposure time of the surgical site tissue, so the more chance of contamination. Mik et al. [47] found that a total operation time of more than 180 minutes increases the risk of surgical site infection in deep incisions and organ spaces. At the same time, the longer the operation time, the greater the possible trauma and the more blood loss, which further reduces the patient ‘s resistance and makes the patient more prone to infection. It is suggested that for patients with long expected operation time, a detailed surgical plan should be formulated before operation, so as to shorten the operation time as much as possible while ensuring the quality of operation, and at the same time, second-generation antibiotics should be given appropriately for prevention and control. This model can not only explain the relationship between features and risk factors but also predict the importance of features for individuals. If the model is prospectively validated, it can help clinicians determine which part of the intervention is most important, thus providing an interpretable and powerful tool for preventing postoperative infection.

This study has several remarkable limitations. First of all, the training sample size is limited, because the queue only comes from one center, which may lead to over-fitting of the model. In the future, multi-center research is needed for external verification. Secondly, this study is retrospective, and there may be collection and input bias and inevitable selection bias. For example, the incidence of postoperative anastomotic leakage is extremely low in our study. To improve the performance of artificial intelligence models, the models established in this study will eventually be applied to other medical sites to verify their scalability.

In summary, our study demonstrates for the first time that the MGA-XGBoost model with 10 risk factors can predict postoperative infectious complications in elderly CRC patients. At the same time, combining risk prediction with feature importance analysis allows clinicians to assess postoperative risks and potentially modifiable drivers.

## Data Availability

The data used to support the findings of this study are available from the corresponding author upon reasonable request.
